# Clinical Molecular Pathology and Treatment Developments in Advanced Uveal Melanoma: State of the Art

**DOI:** 10.32604/or.2025.071831

**Published:** 2026-01-19

**Authors:** Stefano Dore, Matteo Sacchi, Antonio Pinna, Giuseppe Palmieri, Panagiotis Paliogiannis

**Affiliations:** 1 Unit of Ophthalmology, Department of Medicine, Surgery and Pharmacy, University of Sassari, Viale San Pietro 43, Sassari, 07100, Italy; 2 Unit of Cancer Genetics, Institute of Genetic & Biomedical Research (IRGB), National Research Council (CNR), Traversa La Crucca n. 3, Sassari, 07100, Italy; 3 Immuno-Oncology & Targeted Cancer Biotherapies, University Hospital (AOU) of Sassari, Viale San Pietro 43, Sassari, 07100, Italy; 4 Anatomic Pathology and Histology, University Hospital (AOU) of Sassari, Via Matteotti 60, Sassari, 07100, Italy

**Keywords:** Eye, uveal melanoma (UM), molecular pathology, targeted therapy, immunotherapy

## Abstract

Uveal melanoma (UM) is the most common intraocular cancer, with approximately 5.2 individuals per million affected annually in the United States. It represents approximately 3% of the global malignant melanoma cases, accounting for 80% of the overall noncutaneous melanomas. Clinically, it remains silent in about 30% of the cases; when symptomatic, it generally causes metamorphopsia (painless loss or distortion of vision) and/or photopsia (flashing or flickering of light in the visual field). Discoloration of the iris, astigmatism, glaucoma, and even blindness are other, less common clinical manifestations. Several pathophysiological mechanisms underlie the development of UM. Genetic mutations, involving especially the G protein subunit alpha q (GNAQ), guanine nucleotide-binding protein subunit alpha-11 (GNA11), BRCA1 associated deubiquitinase 1 (BAP1), splicing factor 3b subunit 1 (SF3B1), and eukaryotic translation initiation factor 1A, X-linked (EIF1AX) genes as well as the MAPK/ERK signaling pathway genes, have been largely associated with the development of UM. Chromosomal aberrations, inflammatory and immunological alterations are often concurrent factors for the development and progression of UM. Therapies targeting specific genetic alterations and immunotherapy agents have been recently developed and introduced in clinical practice for the management of advanced-stage UMs. This review aims to present the latest advances in the clinical molecular pathology of UM, along with the resulting targeted, immunological, and other therapies that have been introduced or are currently under investigation.

## 1 Introduction

Malignant melanoma is a highly aggressive neoplasm originating from melanocytes in the basal layer of the epidermis and other tissues, often defined by rapid progression, significant metastatic potential, and elevated mortality despite comprising only approximately 1% of skin cancers [1]. Globally, its incidence continues to rise, with an estimated 325,000 new cases in 2020, which accounts for a 41% increase from 2012. In 2023–2024, the U.S. projected approximately 97,600 new invasive melanoma diagnoses and nearly 8000 deaths [2]. Incidence is highest in fair-skinned populations in Australia, New Zealand, and Western Europe, with ultraviolet (UV) radiation, especially UVB, driving characteristic C > T dipyrimidine transitions (UV-signature mutations) in sun-exposed cutaneous lesions [3].

At the molecular level, genomic profiling, including The Cancer Genome Atlas datasets, has defined four principal subtypes: *BRAF*-mutant (40%–50%, harbouring *V600E/K*), *NRAS*-mutant (15%–20%), *NF1*-mutant (10%–15%), and triple-wild-type, each with distinct therapeutic implications [4]. Additional drivers include *KIT* mutations (particularly in acral/mucosal melanomas), *TERT* promoter alterations, *CDKN2A* inactivation and chromatin regulator defects like BRCA1-associated deubiquitinase 1 (*BAP1*), *MITF*, and *POT1*, associated with familial syndromes [2,5–7]. Epigenetic modifications, such as promoter hypermethylation of *CDKN2A/INK4A*, *SYK* silencing and aberrant histone acetylation/deacetylation, have emerged as key contributors to tumour progression and therapeutic resistance [8].

Because melanocytes are distributed throughout various tissues, malignant melanoma can arise in virtually any anatomical location, irrespective of tissue type. Accordingly, malignant melanoma is broadly classified into cutaneous and non-cutaneous forms. Non-cutaneous melanomas (NCMs) are further subdivided based on their site of origin into acral, ocular, mucosal, and melanomas of unknown primary. Although significantly less common than cutaneous melanoma, ocular and mucosal subtypes account for approximately 5.5% and 1.3%, respectively, of all melanoma cases in North America [9,10]. Ocular melanoma is the most prevalent subtype of NCM and refers to any melanoma originating within the eye. However, the term is most commonly used to denote uveal melanoma (UM), which accounts for the vast majority of ocular melanoma cases. UM arises from melanocytes located in the uvea, the pigmented middle layer of the eye composed of the iris, ciliary body, and choroid. Among these, the choroid is the most frequent site of origin. Ocular melanomas affecting other eye districts, such as those involving the conjunctiva, are significantly rarer and biologically distinct [11].

The age-standardized incidence of UM typically ranges from three to eight cases per million persons per year, depending on geographic region, being highest in Europe (7.3 cases/million) and lowest in East Asia (0.4 cases/million) [12]. In the United States, incidence has remained stable at approximately 5.1–5.2 per million over the past decades [13,14]. Meta-analytical data indicate a modest decline in North America but stable rates in Europe [15]. The median age at diagnosis is typically between 60 and 62 years, with a slight male predominance [16]. Five-year relative survival remains around 60%–67%, influenced by tumour stage and histology [13].

Primary treatment of early-stage UM typically involves globe-sparing radiotherapy, most commonly plaque brachytherapy with iodine-125 or ruthenium-106, laser therapy, or enucleation in cases of large tumours or intractable pain [17]. Despite high local control rates, approximately 50% of patients develop metastatic disease, for which systemic treatment options are necessary. Emerging therapeutic strategies for metastatic UM include MEK and PKC inhibitors, immune checkpoint blockade, along with combinatorial approaches integrating molecular targeting with immunomodulation to overcome tumour immune evasion.

The aim of this review is to present from a clinical perspective the latest advances in the molecular pathology of UM, along with the resulting targeted, immunological and other therapies that have been introduced or are currently under investigation.

## 2 Clinical Manifestations and Workup

Timely recognition of UM’s clinical manifestations is critical for early intervention and prognostic assessment. Clinical presentation is highly dependent on tumour location, size, and secondary effects on ocular structures. Choroidal melanoma, the most frequent subtype, often presents as a dome- or mushroom-shaped pigmented mass detectable on fundoscopy and associated with characteristic features such as subretinal fluid, orange pigment (lipofuscin), localized retinal detachment, and overlying retinal pigment epithelium alterations. Common visual symptoms include blurry vision, photopsia, floaters, visual field defects, and metamorphopsia, although up to 30% of patients may remain asymptomatic, with tumours discovered incidentally during routine ophthalmic examination or imaging studies [18,19].

Iris melanomas, less frequent and typically diagnosed earlier due to their anterior location, may cause visible iris discoloration, ectropion uveae, corectopia, hyphema, and secondary glaucoma resulting from angle invasion. In contrast, ciliary body melanomas are often diagnosed at later stages due to their posterior and obscured location, with presentations including lens displacement, astigmatism, zonular disruption, localized cataract, or elevated intraocular pressure [20].

Diagnostic workup includes ultrasonography, optical coherence tomography, ultrasound biomicroscopy for anterior tumours, and fundus autofluorescence to identify lipofuscin. Fine-needle aspiration biopsy may be employed for cytogenetic and molecular classification. Although fewer than 4% of patients present with metastases at diagnosis, up to 50% develop metastatic disease, predominantly to the liver via hematogenous spread, often years after initial treatment [21].

## 3 Clinically Significant Molecular Alterations in UM

UM is characterized by a distinct genetic profile that differs markedly from cutaneous melanoma. The most common early driver mutations involve G protein subunit alpha q (*GNAQ)* and guanine nucleotide-binding protein subunit alpha-11 (*GNA11) genes*, found in approximately 80%–90% of cases, which encode Gα subunits of heterotrimeric G proteins and lead to constitutive activation of the MAPK and YAP pathways [22]. These mutations are mutually exclusive and considered initiating events in UM genesis. Other genetic alterations correlate with prognosis; *BAP1 gene* mutations or loss, frequently associated with monosomy 3, are found in approximately 45%–50% of tumours and confer a high metastatic risk [23]. Conversely, eukaryotic translation initiation factor 1A, X-linked (*EIF1AX)* and splicing factor 3b subunit 1 (*SF3B1) gene* mutations, typically found in tumours with disomy 3, are associated with more favourable outcomes [24]. *SF3B1* mutations are linked to a late-onset metastatic phenotype, whereas *EIF1AX* mutations generally indicate indolent tumours [25]. These mutations are also mutually exclusive as well, indicating distinct molecular subclasses. Let’s now take a closer look at the role of these genes in the pathogenesis and management of UM.

### 3.1 GNAQ

The *GNAQ* gene is located on chromosome 9q21.2 and encodes the Gαq subunit of heterotrimeric G proteins, functioning as a switch that controls intracellular signaling via guanosine triphosphate (GTP) binding and hydrolysis [26]. In UM, somatic activating mutations, predominantly Q209, occasionally R183, abolish GTPase activity and lock Gαq in the active state [27]. This leads to persistent activation of phospholipase Cβ (PLCβ), generation of second messengers inositol 1,4,5-trisphosphate (IP_3_) and diacylglycerol (DAG), activation of protein kinase C (PKC), and downstream stimulation of MAPK/ERK, PI3K/AKT, and YAP/TAZ pathways, promoting proliferation and survival. *GNAQ* mutations are present in 45%–55% of primary UM and in early nevi, suggesting an initiating oncogenic event [22]. These are mutually exclusive with *GNA11* mutations, defining a key molecular subclass of UM [27]. Importantly, mutant Gαq also activates YAP independently of Hippo signaling, and its interaction with ARF6 contributes to β-catenin nuclear translocation and MAPK resistance mechanisms [28].

### 3.2 GNA11

The *GNA11* gene resides at chromosome 19p13.3 and encodes the Gα11 protein, a paralog of Gαq sharing nearly 90% sequence identity [26]. Mutations typically occur at hotspots Q209 and, less commonly, R183, inactivating intrinsic GTPase function and locking Gα11 in an active GTP-bound state [22,27]. This leads to constitutive PLCβ activation, production of IP_3_ and DAG, and downstream engagement of PKC, MAPK/ERK, PI3K/AKT, and YAP/TAZ signaling, analogous to *GNAQ*-driven signaling [29]. Preclinical models confirm that *GNA11 Q209L/R* mutations induce spontaneous melanoma and metastasis in mice, with robust MAPK activation [22,30]. Although both genes initiate UM, *GNA11* mutations are more frequently associated with high-risk cytogenetics (e.g., monosomy 3, BAP1 loss) and shorter disease-specific survival compared to *GNAQ* mutants (26 vs. 31 months) [31,32].

### 3.3 BAP1

The *BAP1* gene is located on chromosome 3p21 (generally 3p21.1–p21.31) and encodes BRCA1-associated protein-1, a nuclear deubiquitinating enzyme (729 amino acids) integral to chromatin remodelling via the polycomb repressive deubiquitinase (PR-DUB) complex [33]. Its N-terminal ubiquitin carboxy-terminal hydrolase (UCH) domain removes monoubiquitin from H2AK119, modulating gene expression and maintaining differentiation state. *BAP1* loss-of-function mutations or protein loss frequently coincide with monosomy 3 and are strongly associated with early metastasis and poor prognosis (47% of cases; 84% of metastatic UM) [32]. *BAP1* also influences DNA damage response, cell-cycle regulation, stemness, and ER-mediated calcium release via IP3R3 stabilization, contributing to apoptosis promotion. As a tumour suppressor, BAP1 interacts with host cell factor C1 (HCF-1), E2F transcription factors, and chromatin regulators, coordinating cell proliferation checkpoints [34].

### 3.4 SF3B1

The *SF3B1* gene is located on chromosome 2q33 and encodes subunit 1 of the spliceosomal complex SF3b, critical for recognition and proper excision of introns during pre-mRNA splicing [35]. Recurrent heterozygous missense mutations, notably at codon R625, lead to aberrant 3′ splice-site usage and generation of altered transcript isoforms, impacting genes involved in cell cycle, DNA repair, and metabolism [36]. In UM, *SF3B1* mutations occur in 15%–20% of cases and are mutually exclusive with *BAP1* and *EIF1AX* mutations, typically found in disomy-3 tumours associated with intermediate prognosis and late-onset metastasis [37]. Although the full spectrum of mis-spliced targets remains under investigation, *SF3B1*-mutant UM shows distinct gene expression profiles and a slower metastatic course, suggesting prognostic and potential therapeutic implications.

### 3.5 EIF1AX

The *EIF1AX* gene resides on chromosome Xp (Xp22-Xp21 region) and encodes eukaryotic translation initiation factor 1A (eIF1A), a small (17 kDa) component of the 43S pre-initiation complex that scans mRNA and mediates start codon selection and Met-tRNA_i_^met^ recruitment to the ribosome [38]. Missense mutations in the N-terminal region appear in approximately 8%–15% of primary UM and are associated with disomy 3 and excellent prognosis, with very low metastatic risk [24,39]. The precise oncogenic mechanism remains unclear, but it may involve subtle modulation of translation initiation fidelity, start-site selection, altered proteome composition, favouring transcripts conferring growth advantage or interactions with other factors [40]. *EIF1AX*-mutant tumours demonstrate significantly longer disease-free survival compared to other molecular subtypes [39].

### 3.6 Other Genes

In addition to canonical mutations, UM harbours a subset of rarer genetic alterations that may influence tumour phenotype, metastatic potential, and therapeutic response. Activating mutations in *CYSLTR2* (L129Q) and *PLCB4* (D630Y) occur in 1%–5% of primary UM and functionally mimic *GNAQ/GNA11* mutations by activating Gαq signaling pathways, including MAPK and YAP/TAZ [41,42]. These mutations are mutually exclusive with each other and with *GNAQ/GNA11*, suggesting alternative routes of early tumorigenesis [43]. Emerging data have also identified alterations in *TP53*, *CDKN2A*, *PTEN*, and mismatch repair genes such as *MLH1* in subsets of metastatic UM, possibly reflecting genomic instability or therapy-related evolution [25,44]. Moreover, structural variants, such as focal amplifications of *MYC* or losses at 1p36, may contribute to aggressive behaviour [45]. These findings underscore the genomic heterogeneity of UM and support comprehensive molecular profiling, particularly in advanced disease, to identify rare but actionable events. Table 1 summarizes the most common gene mutations in UM with their main clinical and prognostic implications.

**Table 1:** Genetic profile of UM

Gene	Prevalence in UM	Clinical significance
*GNAQ*	40%–50%	Early driver mutation. Found in both low- and high-risk tumours. Not directly prognostic but essential for tumour initiation [22,31,32].
*GNA11*	30%–40%	Mutually exclusive with *GNAQ*. May be more frequently associated with aggressive disease, though not definitively prognostic [22,31,32].
*BAP1*	40%–50% in metastatic tumours	Strong negative prognostic marker. Loss of *BAP1* is highly associated with metastatic risk (especially to the liver) and poor survival. Often linked to chromosome 3 monosomy [23,32].
*SF3B1*	15%–20%	Associated with an intermediate prognosis. Tends to correlate with late-onset metastasis (several years post-diagnosis). Found in tumours with disomy 3 [24,25,37].
*EIF1AX*	8%–15%	Linked to favourable prognosis. Tumours with *EIF1AX* mutations rarely metastasize. Usually found in smaller tumours with favourable genomic features [24,39].
*CYSLTR2*	<5%	Rare alternative to *GNAQ/GNA11* mutations. Oncogenic driver under investigation. Prognostic significance not well established [41,42].
*PLCB4*	1%–2%	Another rare alternative driver to *GNAQ/GNA11*. Clinical impact is unclear due to low frequency [41,42]

Note: Abb: UM: uveal melanoma; GNAQ: G protein subunit alpha q; GNA11: guanine nucleotide-binding protein subunit alpha-11; BAP1: BRCA1 associated deubiquitinase 1; SF3B1: splicing factor 3b subunit 1; EIF1AX: eukaryotic translation initiation factor 1A, X-linked; CYSLTR2: cysteinyl leukotriene receptor 2; PLCB4: phospholipase C beta 4.

### 3.7 Cutaneous Melanoma Driver Genes

Some additional considerations need to be made regarding cutaneous and mucosal melanoma driver genes, like *BRAF*, *NRAS*, and *KIT*, which represent a particular clinical interest as they currently represent specific precision therapy targets. Unlike cutaneous melanoma, in which *BRAF* mutations occur in approximately half of the cases [46,47], *BRAF* mutations are exceedingly rare in UM, occurring typically in tumours located at the iris rather than the choroid or ciliary body. When present, *BRAF* V600E mutations activate the MAPK pathway, but their biological significance in UM remains limited due to its distinct oncogenic landscape. Consequently, some *BRAF* inhibitors, such as vemurafenib, have not demonstrated efficacy in UM patients [21]. Similarly, *NRAS* and *KIT* mutations are exceedingly rare in UM, lacking recurrent hotspot patterns and showing no significant oncogenic or therapeutic relevance [48]. The rarity of these mutations underscores the molecular divergence between UM and cutaneous melanoma, emphasizing the need for pathway-specific therapeutic strategies.

## 4 Targeted Therapies

**Figure 1:**
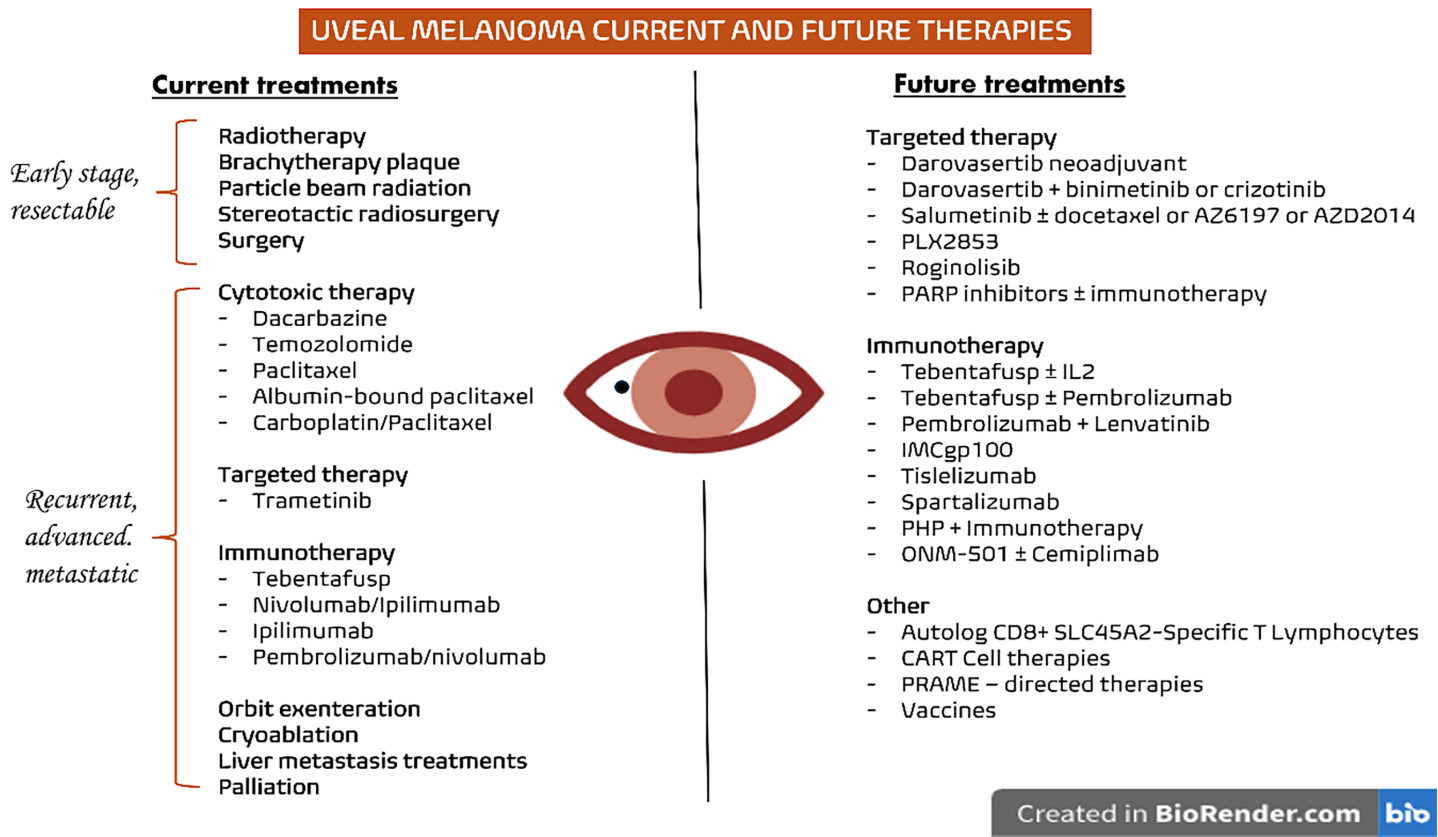
Current and future therapies for UM. On the left, the treatments currently used in clinical practice are summarized according to the disease stage, while on the right, the main therapeutic approaches under investigation for UM are presented. Abb: PARP: Poly(ADP-ribose) Polymerase; PHP: Percutaneous Hepatic Perfusion; CAR-T: Chimeric Antigen Receptor T cells; PRAME: Preferentially Expressed Antigen in Melanoma; IL: Interleukin; SLC45A2: Solute Carrier Family 45 Member 2

Several approaches are currently available for the treatment of early-stage UM, including enucleation, brachytherapy plaque, particle beam radiation, traditional radiation, and others [49]. In cases of unresectable or metastatic disease, systemic treatments are necessary, including cytotoxic, targeted, and immunologic therapies (Fig. 1).

### 4.1 Trametinib

Targeted therapy options are essentially limited to the use of trametinib, considering that *BRAF V600* mutations are very rare in UM, as mentioned above [49]. Trametinib is a selective MEK1/2 inhibitor used primarily in *BRAF V600E/K*-mutated cancers. It disrupts the MAPK/ERK pathway, thereby inhibiting tumour cell proliferation and survival [50]. Clinical efficacy has been demonstrated in melanoma and other solid tumours, though resistance often emerges via reactivation of upstream signaling [51,52]. A randomized phase II study evaluated the efficacy of trametinib alone versus in combination with GSK2141795 (a pan-AKT inhibitor) in patients with metastatic UM. Fifty-seven patients were enrolled. The study found no significant improvement in progression-free survival (PFS) with the combination therapy compared to trametinib monotherapy (median PFS: 1.8 vs. 1.9 months, respectively). Toxicity was higher in the combination arm, with increased rates of rash, diarrhoea, and liver enzyme elevation. These results suggest limited efficacy of dual MEK/AKT blockade in unselected UM patients [53].

### 4.2 Darovasertib

Darovasertib (IDE196) is a selective small-molecule inhibitor of PKC, developed to specifically target oncogenic mutations in *GNAQ* and *GNA11*, which are found in approximately 90% of UM cases, as mentioned above. Inhibition of PKC by Darovasertib has demonstrated preclinical efficacy in reducing ERK phosphorylation and YAP-mediated transcription, thereby suppressing tumour proliferation [22]. *In vitro* studies and *in vivo* murine models have shown significant tumour volume reduction, particularly when Darovasertib is combined with MEK inhibitors such as binimetinib [54]. Preliminary results from the ongoing phase I/II clinical trial report a disease control rate of 89% in metastatic UM patients treated with Darovasertib monotherapy, with even more promising responses observed when combined with crizotinib, a c-MET inhibitor [55,56]. The most frequent adverse events with this combination include gastrointestinal toxicity (nausea, vomiting, diarrhea), fatigue, hepatic enzyme elevation, peripheral edema, hypotension, and rash, with hepatotoxicity and hypotension representing the most relevant grade ≥3 toxicities observed. Globally, the safety profile appears favourable, with most adverse events being manageable and of low severity; nevertheless, tebentafusp toxicity is generally more manageable, with cytokine release syndrome and skin reactions being common but mostly low-grade and predictable. The results of the ongoing phase II/III clinical trial (NCT05987332), which aims to evaluate the efficacy of this drug combination, are expected. In any case, these findings support the concept of PKC as a key oncogenic driver in UM and position Darovasertib as one of the most promising therapeutic candidates in a disease setting historically lacking effective systemic treatments.

### 4.3 Ongoing Trials

Several interesting studies are currently ongoing regarding targeted therapies in UM. The NCT02768766 trial evaluates selumetinib, a selective MEK1/2 inhibitor, in combination with dacarbazine for advanced UM. By targeting the MAPK pathway hyperactivated via *GNAQ/GNA11* mutations, selumetinib aims to enhance anti-tumour efficacy. Preliminary data suggest modest clinical benefit with manageable toxicity [57]. Further investigations into combination therapies revealed that combining selumetinib with docetaxel, the ERK inhibitor AZ6197, or the mTORC1/2 inhibitor AZD2014 resulted in significant tumour growth inhibition in certain UM Patient Derived Xenograft (PDX) models. Combination with paclitaxel in a recent phase II study did not lead to significant clinical outcomes [58].

A phase Ib/IIa trial (NCT03297424) investigates PLX2853, an oral BET (bromodomain and extra-terminal) inhibitor, in patients with advanced solid tumours and lymphoma. The study aims to assess the safety, pharmacokinetics, pharmacodynamics, and preliminary efficacy of PLX2853. Preliminary results indicate that PLX2853 is well-tolerated, with dose-proportional pharmacokinetics and transient target engagement. Clinical activity includes one complete response in diffuse large B-cell lymphoma, two partial responses in UM and primary peritoneal cancer, and stable disease in 14 patients. A randomized phase II registrational trial evaluating roginolisib (IOA-244), a highly selective allosteric PI3Kδ inhibitor, in patients with metastatic UM refractory to standard therapies has been recently registered in Europe (EudraCT: 2024-514333-37-00). The study investigates pharmacodynamic immune modulation, antitumor activity, and safety/tolerability. Finally, PARP inhibitors (PARPi) such as olaparib and niraparib have emerged as potential therapeutic agents in UM, particularly in combination with immune checkpoint inhibitors (ICIs). A phase II trial (NCT05524935) is currently investigating the efficacy of olaparib combined with pembrolizumab in patients with advanced UM. Preclinical studies suggest that PARPi can increase neoantigen load and tumor mutational burden, enhancing the effectiveness of ICIs [59]. Additionally, PARPi have been shown to upregulate PD-L1 expression, potentially increasing immune-mediated tumor cytotoxicity [60]. While early clinical data are limited, these findings support the exploration of PARPi and ICI combinations in UM treatment.

### 4.4 Routine Genetic Profiling in UM Patients

The data reported so far raises the question of the utility of genetic testing in the clinical management of UM patients. Germline genetic testing is not routinely recommended for all patients with UM, but it is advised selectively, mainly when there is suspicion for a hereditary tumor predisposition syndrome such as BAP1 tumor predisposition syndrome, or when there is a strong family history, as this information has preventive value for relatives and for second primary risk, rather than changing current therapeutic decisions for the patient. In contrast, somatic testing (monosomy 3, chromosome 8q gain, BAP1 loss, and gene expression profiling) is useful for metastatic risk stratification and patient counseling and is also of major value for research purposes and for the implementation or enrichment of clinical trials, where molecular profiling is increasingly required. Gene expression profiling is currently used for classifying UM into Class 1 (disomy 3, no chromosome 8q gain, BAP1 preserved) or 2 (monosomy 3, chromosome 8q gain, BAP1 loss of function), which translates into low and high risk for developing metastasis, respectively [61].

The need for molecular profiling of UM brings with it a relevant practical issue concerning the method by which tissue should be obtained to perform molecular testing. Although primary tumor biopsy can provide valuable pathologic information in non-surgical cases, intraocular biopsies remain invasive and carry rare but potentially severe risks (e.g., hemorrhage, retinal detachment, cataract). Moreover, concerns persist among some clinicians about the possibility of systemic tumor spread after biopsy. Finally, biopsies may yield discordant molecular profiles depending on sampling location, meaning that a single biopsy may inadequately capture tumor heterogeneity, and repeated sampling is not feasible or easy to perform [62]. Less invasive and more easily repeatable techniques, such as transvitreal and transscleral fine needle aspiration (FNAB) or liquid biopsy, may be useful for this purpose, also thanks to the major advances in gene sequencing technologies achieved in recent years. A recent meta-analysis including seven studies and 518 cases underscored circulating tumor DNA as a potential predictor of worse survival in patients with metastatic UM, highlighting the potential of liquid biopsy to refine risk stratification and guide treatment strategies [63].

## 5 Immunotherapy

### 5.1 Tebentafusp

Tebentafusp for HLA A*0201-positive tumours has been recently introduced for the treatment of UM. Tebentafusp is part of a class of immune-mobilizing monoclonal T-cell receptor (TCR) therapies designed to target cancer. It is a high-affinity molecule that binds to gp100, a protein highly expressed and presented by HLA-A*0201 on melanoma cells, as well as to the CD3 receptor on T lymphocytes [64]. Its mechanism of action involves robust activation of the immune system, resulting in strong stimulation of both helper (Th) and cytotoxic (Tc) T cells. This leads to an enhanced cytotoxic immune response against tumour cells, with the intensity of the response correlating with the level of gp100 expression on the tumour surface [65]. Early phase clinical trials confirmed that tebentafusp-based therapy is generally well tolerated, and in a phase II trial, the overall response rate (ORR) was 5%, with a reduction in target lesions observed in 44% of patients and a 12-month overall survival (OS) rate between 62% and 86% [66]. Later, a phase III study showed that treatment with tebentafusp resulted in a significantly higher 12-month OS compared to the control group (73% vs. 59%), along with improved PFS at 6 months (31% vs. 19%) [67].

### 5.2 ICIs

Regarding ICIs, ipilimumab, a fully human monoclonal antibody targeting cytotoxic T-lymphocyte-associated protein 4 (CTLA-4), has been extensively studied in cutaneous melanoma; however, its efficacy in UM remains limited [68]. This is because, unlike cutaneous melanoma, UM has a low tumour mutational burden and a distinct immunobiology characterized by poor T-cell infiltration and immunosuppressive microenvironment, contributing to resistance to ICIs [25]. Clinical studies of ipilimumab monotherapy in metastatic UM have shown modest benefit, with ORR typically below 5% and median PFS of approximately 2.8 months [69]. In a multicentre retrospective analysis, ipilimumab (3 mg/kg or 10 mg/kg) yielded disease control in only a minority of patients, with significant immune-related toxicities such as colitis, diarrhea, hepatitis, dermatitis, rash with pruritus, and endocrine disorders like hypophysitis and thyroid dysfunction, with liver toxicity being especially clinically relevant because of frequent hepatic disease involvement in this tumor type [70]. Combination immunotherapy with ipilimumab and nivolumab, a monoclonal antibody targeting the programmed cell death-1 (PD-1) receptor, has demonstrated slightly improved outcomes, albeit at the cost of increased toxicity [71,72]. Despite suboptimal results, ipilimumab remains a component of therapeutic strategies in UM due to its potential to synergize with other immunomodulatory agents. Ongoing studies aim to improve efficacy through combination regimens or by overcoming immune exclusion and liver-specific immune tolerance [73].

Other immunotherapy agents used in UM are pembrolizumab and nivolumab, anti-PD 1 monoclonal antibodies, which have revolutionized the treatment of cutaneous melanoma but show limited efficacy in UM. Monotherapy with pembrolizumab or nivolumab has demonstrated ORR of <5%, with a median PFS ranging from two to three months in metastatic UM [74,75]. However, some durable responses have been observed in a subset of patients, especially those with low tumour burden and limited hepatic involvement [76]. A recently published retrospective analysis of data from 131 metastatic UM patients treated with ipilimumab and nivolumab in five centers in Switzerland from 2016 to 2024, showed that those with exclusively extrahepatic metastases had a better ORR and OS compared to those with hepatic or mixed metastases (*p* = 0.02, *p* = 0.02, respectively). Interestingly, 20.6% of patients developed eosinophilia during treatment, which was associated with improved median OS (24 months vs. 15 months, *p* = 0.02) [77].

Regarding the oncological outcomes, a metanalysis of data from five databases evaluating the outcomes of ipilimumab alone or in combination with other ICIs reported that pooled ORR was 9.2% overall (95% CI 7.2–11.8) [4.1% for anti-CTLA4 (95% CI 2.1–7.7), 7.1% for anti-PD(L)1 (95% CI 4.5–10.9) and 13.5% for anti-CTLA4 plus anti-PD1 (95% CI 10.0–18.0)]. Median OS was 11.5 months overall (95% CI 9.5–13.8) [8.0 months for anti-CTLA4 (95% CI 5.5–9.9), 11.7 months for anti-PD(L)1 (95% CI 9.0–14.0) and 16.0 months for ipilimumab plus anti-PD1 (95% CI 11.5–17.7) (*p* < 0.001)]. Median PFS was 3.0 months overall (95% CI 2.9–3.1) [78].

A more recent metanalysis including 36 articles representing 41 cohorts of 1414 patients with metastatic UM, evaluated the outcomes of single versus dual ICI treatments. Compared to single-agent, dual ICI led to better ORR (single-agent: 3.4% [95% CI 1.8–5.1]; dual-agent: 12.4% [95% CI 8.0–16.9]; *p* < 0.001), DCR (single-agent: 29.3%, [95% CI 23.4–35.2]; dual-agent: 44.3% [95% CI 31.7–56.8]; *p* = 0.03), and OS (single-agent: 9.8 months [95% CI 8.0–12.2]; dual-agent: 16.3 months [95% CI 13.5–19.7]; *p* < 0.001) [79].

Despite the limited potential of ICIs in the treatment of UM, several clinical trials including immunotherapy agents are currently ongoing. In these studies, immunotherapeutic agents are usually tested in combination with each other or with other therapeutic approaches, such as the different techniques used for the treatment of liver metastases, which as previously discussed, represent a major limiting prognostic factor. There are several direct and indirect strategies for treating hepatic metastases, ranging from surgery to ablative techniques (radiofrequency or microwave), embolization procedures (transarterial chemoembolization, immunoembolization, radioembolization), and percutaneous isolated hepatic perfusion (PHP). Table 2 summarizes the most relevant trials currently registered in the USA and Europe.

**Table 2:** Ongoing clinical trials testing immunotherapy agents in UM patients registered in the USA and the European Union

Trial ID	Phase	Country	Treatment	Indication
NCT05077280	2	USA	Stereotactic Body RT + Opdualag	Metastatic UM
NCT04283890	1/2	Netherlands	PHP + Ipilimumab-Nivolumab	Metastatic UM
NCT07063875	1/2	Australia	Tebentafusp + IL-2	Metastatic UM
NCT03070392	2	International	IMCgp100 vs. investigator’s choice	HLA-A*0201+ patients with untreated advanced UM
NCT06519266	3	Sweden	PHP + Ipilimumab/Nivolumab vs. Ipilimumab/Nivolumab	Metastatic UM
NCT03068624	1	USA	Autologous CD8+ SLC45A2-Specific T Lymphocytes + Cyclophosphamide, Aldesleukin, and Ipilimumab	Metastatic UM
NCT03635632	1	USA	C7R-GD2.CAR-T Cells	Neuroblastoma and other GD2 positive cancers
NCT04802876	2	Spain	Tislelizumab and Spartalizumab	Multiple cancers with PD1-high MRNA expression
NCT03686124	1	USA/Germany	ACTengine^®^ IMA203/IMA203CD8 ± Nivolumab	Recurrent—refractory solid tumours
NCT06022029	1	USA/Australia	ONM-501 ± Cemiplimab	Advanced solid tumours and lymphomas
2024-514657-30-00	2	France	Pembrolizumab + Lenvatinib	Metastatic UM
2024-517290-24-00	2	International	IMCgp100 vs. investigator’s choice	HLA-A*0201+ patients with untreated advanced UM
2022-502732-39-00	2/3	International	Tebentafusp ± Pembrolizumab vs. investigator’s choice	HLA-A*0201+ previously treated advanced melanoma
2023-508156-20-00	3	Sweden	PHP + Ipilimumab/Nivolumab vs. Ipilimumab/Nivolumab	Metastatic UM
2024-516127-14-01	1	Netherlands	PHP + Ipilimumab/Nivolumab	Advanced UM

Note: Abb: PD1: programmed death 1; PHP: Percutaneous Hepatic Perfusion; RT: radiotherapy; UM: uveal melanoma; IL: interleukin; CAR-T: Chimeric Antigen Receptor T cells.

## 6 Future Perspectives

As previously discussed, therapeutic options for advanced UM remain extremely limited, both in terms of availability and clinical efficacy. For this reason, the current National Cancer Comprehensive Network guidelines recommend patients with UM to be treated in specialized centres and to be included, when possible, in ongoing clinical trials [49]. Fig. 1 depicts the current treatment landscape of UM, along with the most interesting research efforts for the identification of novel therapies currently ongoing. Several trials are, in fact, investigating alternative treatment approaches, with their results being highly expected in the near future.

### 6.1 Neoadjuvant Treatments

All the treatments described above have been used in the adjuvant setting, but to date, none of them has demonstrated a clear survival benefit in prospective randomized studies; this represents still one of the primary objectives for future studies in the field of UM. Another interesting topic under active investigation is the implementation of neoadjuvant therapy in specific subsets of patients with UM. Neoadjuvant therapy aims to reduce primary ocular tumour burden prior to definitive local treatment. In an investigator-sponsored trial, monotherapy with darovasertib administered before plaque brachytherapy or enucleation induced a greater than 20% tumour shrinkage in 59% of patients and enabled eye preservation in 61%, with acceptable safety [80]. A case report described 80% reduction in tumour size after darovasertib plus crizotinib, allowing preservation of vision in a patient who would otherwise require enucleation [81]. These findings provide proof-of-concept for neoadjuvant systemic therapy in primary UM and support ongoing phase II trials (NCT05907954, NCT05187884, EUCT2023-506683-14-00) to evaluate organ preservation and functional outcomes.

### 6.2 Chimeric Antigen Receptor T (CAR-T) Cell Therapy

Another topic under investigation is the use of chimeric antigen receptor T (CAR-T) cell therapy in UM. Preclinical studies have demonstrated that HER2-directed CAR-T cells eradicate UM xenografts, including tumours resistant to TIL therapy, in human IL-2 transgenic NOG mouse models [82]. More recently, inducible caspase-9 (iCas9) B7-H3-targeted CAR-T cells achieved durable eradication of liver metastases in murine UM models and are advancing toward Phase I clinical evaluation [83]. TYRP1-targeted CAR-T constructs also demonstrated potent antigen-specific cytotoxicity against UM cell lines *in vitro* and *in vivo* without ocular toxicity [84]. These findings validate CAR-T strategies as a promising platform to overcome UM’s immunosuppressive microenvironment and antigen heterogeneity, providing the rationale for further translational efforts.

### 6.3 Preferentially Expressed Antigen in Melanoma (PRAME)-Guided Therapy

Encouraging results were furthermore obtained regarding the Preferentially Expressed Antigen in Melanoma (PRAME)-guided therapy. PRAME is frequently overexpressed (50%–70%) in metastatic UM, co-expressed with HLA-A02, providing a clinically actionable target. *In vitro*, PRAME-specific T cells recognize and kill PRAME-expressing UM lines in an HLA-A02-restricted manner [85]. The ongoing Phase 1b IMA203 (BPX-701, NCT02743611) trial demonstrated a confirmed objective response rate of 67% and a median PFS of 8.5 months in heavily pretreated UM patients, with durable responses up to more than eleven months and acceptable safety. Additionally, ISA103, a PRAME peptide vaccine combined with checkpoint blockade, is under testing to evaluate immunogenicity and response. Concerning vaccines, it is important to mention the phase I trial NCT04335890, which investigates RNA-loaded, IKKβ-matured dendritic cell vaccines in metastatic UM. This autologous cellular immunotherapy aims to enhance antigen-specific T cell responses against tumour-associated antigens. The study evaluates safety, immunogenicity and feasibility of repeated intradermal DC vaccinations in combination with immune checkpoint inhibition and may represent the cornerstone of a significant shift in the future management of advanced UM.

## 7 Conclusions

Advanced UM is a relatively rare malignancy, yet it remains highly challenging to treat. For this reason, patients with UM should be treated in specialized centres and enrolled in clinical trials. Traditional chemotherapy has shown minimal efficacy, and both targeted and immunotherapeutic strategies are limited, especially in terms of lack of meaningful efficacy, minimal impact on overall survival and the short-lived nature of the few responses. These shortcomings reflect the profound biological resistance of this disease and underscore the urgent need for continued research and clinical innovation. Somatic genetic testing through traditional or modern sampling methods is advisable, when possible, for metastatic risk stratification and patient counselling, as well as for research purposes and for the implementation or enrichment of clinical trials. Several ongoing clinical trials are currently exploring novel treatment combinations aimed at overcoming the limitations of monotherapy. Furthermore, emerging therapeutic strategies, including neoadjuvant protocols, CAR-T cell therapies, PRAME—guided treatments, and tumour-specific vaccines are under investigation, with the goal of addressing the current lack of effective options for patients with advanced UM.

## Data Availability

Not applicable.
